# Plant volatiles as cues and signals in plant communication

**DOI:** 10.1111/pce.13910

**Published:** 2020-10-26

**Authors:** Velemir Ninkovic, Dimitrije Markovic, Merlin Rensing

**Affiliations:** ^1^ Department of Ecology Swedish University of Agricultural Sciences Uppsala Sweden; ^2^ Department of Crop Production Ecology Swedish University of Agricultural Sciences Uppsala Sweden; ^3^ Faculty of Agriculture, University of Banja Luka Banja Luka Bosnia and Herzegovina

**Keywords:** adaptation, competition, cues, defence, herbivory, pathogens, signalling, volatile emissions

## Abstract

Volatile organic compounds are important mediators of mutualistic interactions between plants and their physical and biological surroundings. Volatiles rapidly indicate competition or potential threat before these can take place, and they regulate and coordinate adaptation responses in neighbouring plants, fine‐tuning them to match the exact stress encountered. Ecological specificity and context‐dependency of plant–plant communication mediated by volatiles represent important factors that determine plant performance in specific environments. In this review, we synthesise the recent progress made in understanding the role of plant volatiles as mediators of plant interactions at the individual and community levels, highlighting the complexity of the plant receiver response to diverse volatile cues and signals and addressing how specific responses shape plant growth and survival. Finally, we outline the knowledge gaps and provide directions for future research. The complex dialogue between the emitter and receiver based on either volatile cues or signals determines the outcome of information exchange, which shapes the communication pattern between individuals at the community level and determines their ecological implications at other trophic levels.

## Abstract 2

Released volatiles provide information about the identity and physiological status of neighbouring plants. The unique volatile profiles and release of specific cues and signals are guiding plants to response in a timely manner to cope with upcoming challenging threat, stress and to adapt to neighbours.

## 1 INTRODUCTION

Plants coexist in different communities wherein they unavoidably interact with neighbouring plants. Consequently, plants have adapted a variety of strategies to rapidly exchange volatile messages and mitigate the impact of complex biotic and abiotic factors from their surroundings (Douma, Vermeulen, Poelman, Dicke, & Anten, 2017; Vicherova, Glinwood, Hajek, Smilauer, & Ninkovic, 2020). One adaptation mechanism that plants have developed is the ability to produce and emit an array of different volatile organic compounds (VOCs) (Dudareva, Klempien, Muhlemann, & Kaplan, 2013), presenting a fingerprint of the plant's current physiological status. One of the primary functions is to mediate interactions with mutualists such as beneficial microbes, pollinators and seed dispersers, while another function is to deter harmful interactions with herbivorous insects or pathogens (Baldwin, 2010; Caruso & Parachnowitsch, 2016; Chen et al., 2020; Pangesti et al., 2016; Paré & Tumlinson, 1999; Xu & Turlings, 2018). Neighbouring plants use VOC cues and signals to compete for limited resources, enhance direct resistance against herbivores, microbes, and fungi, or to attract predators (Hammerbacher, Coutinho, & Gershenzon, 2019; Ninkovic, Markovic, & Dahlin, 2016; Turlings & Erb, 2018).

The effectiveness of volatile communication between neighbouring plants is context‐dependent and is determined by the plants involved. The specific stress encountered by the emitter plant is reflected through a blend of unique volatiles that can trigger activation or suppression of different genetically encoded programmes (known as priming) and pathways in the receiver plants to prepare a response that will match the upcoming stress (Conrath, Beckers, Langenbach, & Jaskiewicz, 2015; Martinez‐Medina et al., 2016). However, this is not always the case, as some herbivorous insects have developed mechanisms to manipulate VOC biosynthesis by plants to send deceptive signals that mislead the receiver to make wrong decisions, from which the manipulator can benefit (Zhang et al., 2019). Therefore, deciphering of specific VOC messages from among all surrounding noises by exposed plants, followed by their unique finely tuned responses, demonstrate the important role VOCs play in coordinating plant response to stress at the community level. Here, we review the ecological role of plant volatile cues and signals in plant interactions and highlight the molecular mechanisms responsible for their perception by and interplay of receivers at higher trophic levels.

## 2 PLANT–PLANT INTERACTIONS FROM AN ECOLOGICAL PERSPECTIVE

### 2.1 Volatiles as cues and signals

Plant coexistence at the community level requires highly sophisticated interactions between members, where VOCs present a rapid and reliable means of communication. The capacity of receivers to respond to specific VOCs released by emitters can significantly minimise fitness costs and avoid unnecessary and costly responses (Erb, 2018). Growth and development are determined by the genetic potential of plants and their interaction with highly dynamic environmental stimuli. Plants have developed sophisticated mechanisms to detect, perceive and respond to fluctuating VOCs from their surroundings to withstand different environmental challenges and evade their negative impacts (Covarrubias, Cuevas‐Velazquez, Romero‐Perez, Rendon‐Luna, & Chater, 2017).

**FIGURE 1:**
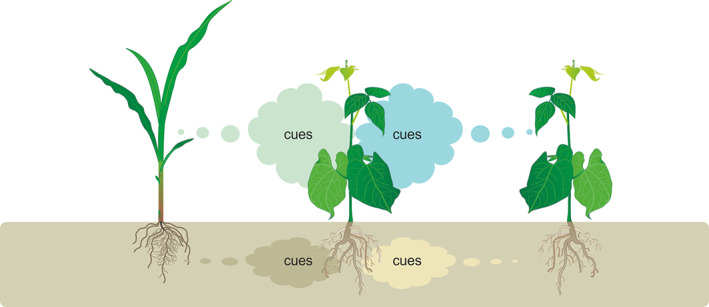
Complexity in below and aboveground plant–plant interactions based on volatile cues. Constitutively released volatile cues provide information about the genetic identity of plants, which are used by individual plants in the process of detection, discrimination and adaptation to competitive neighbours [Colour figure can be viewed at wileyonlinelibrary.com]

**FIGURE 2:**
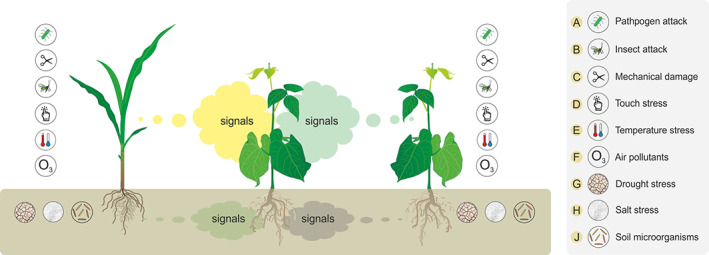
Below and aboveground induced volatile signals released by plants after: A, pathogen attack; B, insect attack; C, mechanical damage; D, touch stress; E, temperature stress; F, air pollutants, G, drought stress; H, salt stress or J, soil microorganisms. Biotic and abiotic stress encountered by plants make them switch to a unique volatile profile and induce the release of specific signals, guiding the receiver to respond in a timely manner to cope with upcoming threat/stress. Simultaneous exposure to the combination of different volatile signals adds to the complexity of plant–plant interactions [Colour figure can be viewed at wileyonlinelibrary.com]

When discussing the role VOCs play in plant–plant interactions, we must distinguish between cues and signals ‐ both from the emitting and the receiving side. Volatile cues are information used by a receiver that is not intentionally released by emitters for that purpose (Figure 1). Volatile signals are intentionally released as a result of the emitters' response to external events with the aim of communicating with the receiver for specific purposes (Figure 2). Constitutively released VOCs carry fingerprint information about the identity of the emitter, which may or may not be used in detecting and adapting to competitive neighbours (Ninkovic et al., 2016). This qualifies the constitutively released VOCs as cues. For instance, certain barley (*Hordeum vulgare*) cultivars exposed to volatiles from another plant allocated more biomass to the roots (Ninkovic, 2003). Parasitic plants can explore constitutively released volatiles that guide them to grow in the direction of their host. In such a way, seedlings of dodder (*Cuscuta pentagona*) can distinguish volatiles from tomato (*Lycopersicon esculentum*) (host) and wheat (*Triticum aestivum*) (non‐host) plants and preferentially grow towards tomato plants because of specific compounds released such as β‐phellandrene, β‐myrcene and α‐pinene (Runyon, Mescher, & De Moraes, 2006). It remains unclear whether parasitized plants change the volatile profile and how these changes affect parasitic plants and conspecific neighbours.

In nature, plants are simultaneously exposed to many different cues and signals that can significantly affect or change the physiological profile of the emitter and, consequently, modify the outcome of their interaction with neighbouring plants. The switch from cue to signal occurs when biotic and abiotic factors change the composition of the emitter VOC profile, which conveys specific intentional information used to communicate with neighbours (Figure 2). For example, in the same barley cultivar combination mentioned above (Ninkovic, 2003), the emitter response to red: far red light was activation of the elongation of the above‐ground part and change in the VOC emission that induced the same shade avoidance response in the receivers (Kegge et al., 2015).

Touch is one way in which the interactions between plants can change volatile emissions that serve as reliable signals to conspecific neighbours. Wind flow is another factor that can entail touch between plants, which is also associated with the heat exchange rate and transpiration rate. In this case, it is important to distinguish the effects of touch caused by wind that change the microclimate from those that are pure touch‐induced mechanical stress (Anten, Alcalá‐Herrera, Schieving, & Onoda, 2010). Plants respond to touch by rapid changes in the emission of volatiles that prime neighbouring plants (Markovic et al., 2019) (Figure 2D). Exposure to touch‐induced volatiles activated the same early defence‐related genes in the neighbouring plants as are induced by touch in the emitter plant, showing that exposed plants could rapidly and accurately mirror the specific changes in the emitter plants. Therefore, it is feasible that the switch from the cue to the signal and vice versa can significantly alter the way in which plants send and perceive volatile messages.

Rapid adaption of plants to changes in the volatile emission of their neighbours demonstrates the important ecological role of touch‐induced VOCs as effective signals for both growth and defence synchronisation at the community level (Elhakeem, Markovic, Broberg, Anten, & Ninkovic, 2018; Markovic et al., 2019). Bearing in mind that any late response to the presence of competitors could be costly may explain why receivers rapidly modify their growth strategy when perceiving specific signals about the changed physiology of their neighbours. A finely tuned response strategy to specific VOCs is a result of processing diverse infochemicals, either single individual compounds or their specific mixture in specific proportions (see *the perception of VOCs by neighbouring plants*). Further studies conducted under field conditions are required to understand how plants discriminate between constitutive cues and periodical signals and how they use the available information during plant–plant interactions.

### 2.2 Floral volatiles in plant communication

In addition to the direct role of constitutively released floral volatiles in pollination and mating, they can function as cues that directly influence the growth of neighbouring plants. For instance, methyl benzoate released by snapdragon flowers (*Antirrhinum majus*) induced response in neighbouring *Arabidopsis thaliana*, reducing root growth (Horiuchi et al., 2007). Considering that stressed plants release floral volatiles in much higher amounts than leaf volatiles (Ibrahim, Egigu, Kasurinen, Yahyab, & Holopainen, 2010), therefore, floral volatiles have been proposed to be used by conspecific neighbours to synchronise flowering because of their highly specific and informative value (Burdon, Raguso, Kessler, & Parachnowitsch, 2015). This assumption has been tested, but evidence on the role of floral volatiles in synchronising flowering among self‐incompatible conspecifics has not been found (Fricke, Lucas‐Barbosa, & Douma, 2019).

Herbivore attacks to other parts of the plant or drought can change the composition of floral volatiles (Burkle & Runyon, 2016; Ramos & Schiestl, 2020), which provide additional information regarding changes in the physiological status of the emitter plant (Figure 2B,G). Moreover, it remains unclear whether the neighbouring plants prioritise between floral or leaf volatiles, and which are more important for adaption responses.

### 2.3 Root volatiles in plant interactions

VOCs play important ecological roles in plant–plant interactions aboveground, raising questions regarding the role of VOCs in root–root interactions (Schenkel, Lemfack, Piechulla, & Splivallo, 2015). The composition of volatile blends released by roots depends on the plant genotype and sex and is characterised by compounds belonging to diverse chemical groups (Delory, Delaplace, Fauconnier, & du Jardin, 2016; Dong, Li, Liao, Chen, & Xu, 2017; Kindlovits et al., 2018). Root‐emitted volatiles provide an additional source of information on the process of detection of competitive neighbours, adding another level to the complexity in plant interactions (Delory et al., 2016). Based on perceived VOCs, plants can differentiate themselves from non‐self roots and sense the degree of genetic relatedness among different root parts (Chen, During, & Anten, 2012; Depuydt, 2014). The fact that 100 different plant species can release (−)‐loliolide and jasmonic acid (JA), which trigger biochemical responses in wheat, demonstrate the existence of a precise mechanism for detecting and responding to the presence of neighbouring plants (Kong et al., 2018).

Constitutively released root‐specific sesquiterpene VOCs of spotted knapweed (*Centaurea stoebe*) promoted the growth and germination of their sympatric neighbours (Gfeller et al., 2019). Belowground plant communication among genetically related individuals can also be mediated by aboveground stress to which neighbours are exposed. Young maize seedlings have shown the capacity to rapidly distinguish neighbour cues from signals and orientate root growth away from signals that are pointing to a stressful environment. High sensitivity to specific signals showed that young plants actively use surrounding messages and participate in interactions with nearby plants (Elhakeem et al., 2018).

The rhizosphere is a complex ecosystem and is crucial for a plant's success, where root‐released VOC plays an important role in root interactions and soil microbiome (Sharifi & Ryu, 2018). Volatiles emitted by roots can act as anti‐microbial or anti‐herbivore substances and contribute to belowground plant defence (Schenkel et al., 2015). For instance, the migration pattern of soil bacteria after the emission of root VOCs changed, and some VOC compounds were able to diffuse over long distances, attracting a variety of soil bacteria (Figure 2J). After infection with a fungal pathogen, the VOC emission profile of the plant changed in a way that it attracted specific bacteria with antifungal properties (Schulz‐Bohm et al., 2018). Belowground interactions between plants and microbes affected the photosynthetic rates and changed the phytohormone signalling pathways (Tyagi, Mulla, Lee, Chae, & Shukla, 2018). The exact molecules involved in the interactions remain unknown, but by changing the rhizosphere in a beneficial or reactive way, consequently, plants will effectively change their VOC profile aboveground, providing another way of possible plant–plant interactions. Taking a look at the aboveground levels of VOC emission and usage from neighbours, it is possible to say that the network of interactions belowground extends to a plant–plant level as well.

### 2.4 Induced responses via volatile cues and effects on higher trophic levels

VOC released by undamaged plants has been shown to have significant implications for the receiving plants and organisms at higher trophic levels (Figure 1), which has previously been termed “allelobiosis” (Ninkovic, Glinwood, & Pettersson, 2006; Pettersson, 2003). The headspace of potato (*Solanum tuberosum*) exposed to onion (*Allium cepa*) contained four times higher concentrations of (E)‐nerolidol and (3E,7E)‐4,8,12‐trimethyl‐1,3,7,11‐tridecatetraene, which had a deterrent effect on green peach aphids (*Myzus persicae*) (Ninkovic et al., 2013). Similar effects on bird cherry‐oat aphids (*Rhopalosiphum padi*) were observed in barley when interacts with specific weeds. Of the 20 most prevalent weed species in cereals, only exposure to volatiles from lamb's quarters (*Chenopodium album*), black nightshades (*Solanum nigrum*) and thistles (*Cirsium arvense* and *Cirsium vulgare*) reduced aphid acceptance of exposed barley plants (Glinwood, Ninkovic, Pettersson, & Ahmed, 2004; Ninkovic & Åhman, 2009). Further exposure to root exudates from couch grass (*Elytrigia repens*) or charlock (*Sinapsis arvensis*) induced the same effect in barley (Dahlin & Ninkovic, 2013; Glinwood et al., 2003). The response to VOCs may influence neighbouring plants to change their root exudate composition and may affect root colonisation by soil microorganisms that are associated with resistance to soil‐borne pathogens (Ehlers et al., 2020; Khashi u Rahman, Zhou, & Wu, 2019). The below and aboveground interactions with certain weeds considerably reduced aphid performance on barley plants, confirming that effective chemical communication occurs only in specific species combinations.

The composition of plant volatile profiles and their total amount is genotype‐dependent and is altered by herbivore or pathogen attacks (Busko, Goral, Boczkowsk, & Perkowski, 2019; Degen, Dillmann, Marion‐Poll, & Turlings, 2004). It has also been shown recently that there are differences in constitutive volatile emissions between genotypes within species (Dahlin, Rubene, Glinwood, & Ninkovic, 2018). These differences may act as preconditions for volatile interactions between specific genotypes. Different barley cultivars experienced stronger induction of defences when receiving VOC cues from specific neighbours, reducing their aphid acceptance (Ninkovic & Åhman, 2009) and population development (Dahlin et al., 2018). However, these effects most frequently occurred in combination with old and modern cultivars, indicating that plant breeding strategies modified the response capacities of receivers (Kellner, Brantestam, Ahman, & Ninkovic, 2010).

In the first instance, higher induction by younger cultivars most likely occurred because they were more genetically homogenous than older cultivars, emitting a more narrow volatile spectrum that gave a clearer but less diverse cue. Second, modern cultivars emitted cues that were more conspicuous to other plants, resulting in a stronger response (Kellner et al., 2010). The applications of these considerations in breeding could be explored in order to restore the desirable properties related to VOC emission or responsiveness acquired by the cultivars' ancestors (Palmgren et al., 2015). However, it is unknown whether this trend could occur with wild plant species, and which mechanisms of genetically related VOC communication will be favoured by natural selection.

In specific plant genotype and species mixtures, exposure of one barley genotype to volatile cues from the other genotype/species caused the VOCs of the exposed plants to become more attractive to seven‐spot ladybirds (*Coccinella septempunctata*). In the same genotype mixtures tested in the field, the ladybird occurrence was significantly higher than that in the pure stands even before the aphid arrival (Ninkovic, Al Abassi, Ahmed, Glinwood, & Pettersson, 2011; Ninkovic & Pettersson, 2003). Similar effects on natural enemies have been observed in mixtures of specific soya genotypes (Grettenberger & Tooker, 2020). Such preferences of natural enemies towards VOCs of exposed plants support the hypothesis that plant volatiles affect their foraging behaviour and that plant genotype mixing shapes the interactions within multitrophic communities. Volatile interactions between some plant species, such as interactions between onions and potatoes, can also change the volatile profile of exposed plants, making them more attractive to ladybirds, thus, contributing to an increased abundance of natural enemies in complex plant habitats (Vucetic et al., 2014).

### 2.5 Interference of biotic factors in plant VOC communication

The production of VOCs is complex and is constantly altered by interactions between plants and biotic factors (Landi, 2020; Pichersky, Noel, & Dudareva, 2006) (Figure 2). Plant–plant communication and the perception of VOCs are mostly studied in systems where emitting plants are under attack by herbivores. Herbivory attack or mechanical damage can rapidly activate plant defence responses, which include changes in the emission of specific VOCs that provide considerable information about the potential threat to neighbours (Coppola et al., 2017; Pezzola, Pandolfi, & Mancuso, 2019).

Detecting plants in the vicinity that are under herbivory attack can give a receiver plant an advantage (Orrock et al., 2015). In this sense, herbivore‐induced volatiles released from an attacked leaf serve as accurate and reliable plant messages for the rapid spread of warning signals to distant parts of the same plant (Heil & Karban, 2010) and for the attraction of natural enemies of the herbivores (Dicke & Baldwin, 2010). Neighbours “eavesdrop” those VOCs to induce a range of ecological and physiological responses and to prepare for defence in a timely manner (Heil & Karban, 2010) (see *the perception of VOCs by neighbouring plants*).

The relatively quick danger posed by moving insects requires plants to mount a response in a similarly quick fashion. Depending on the day‐night cycle, plants release VOCs in a different manner, which indicates the importance of periodic cues (Barrios‐Collado et al., 2016). A clear periodic pattern of changes in VOC release has been observed through diurnal as well as seasonal studies (Patokoski et al., 2015). Periodic changes in VOC emissions could provide a reliable signal for surrounding plants. Rapid plant responses, wherein changes in VOC emission can be observed within minutes (Barrios‐Collado et al., 2016), hold the potential for signals that are eavesdropped, changing the growth and defence strategies. Similar effects can be observed when plants are touched lightly and briefly. Maize (*Zea mays*) responded by the upregulation of defence genes as well as a change in volatile emissions (Markovic et al., 2019). Here, plant–plant communication comes into play, showing that plants receiving these changed VOCs rapidly upregulated the same defence genes as the touched plants.

Plants can differentiate kin from stranger in competitive environments, which is an important trait that promotes cooperation among genetically related individuals (Dudley, Murphy, File, & Robinson, 2013). To prevent strangers from decoding and interpreting specific VOC information that they exchange, kin uses volatile chemotypes as an efficient barrier (Karban, Shiojiri, Ishizaki, Wetzel, & Evans, 2013). For instance, sagebrush (*Artemisia tridentate*) exposed to volatiles from damaged neighbours of the same chemotype triggered a much stronger antiherbivore defence than when exposed to volatiles from different chemotypes (Karban et al., 2014). High sensitivity in the discrimination of VOC chemotypes helps kin support one another and fine‐tune their responses when they encounter stressful situations. Such a pattern in VOC exchange highlighted the importance of discrete communication among plants at the family level (Karban et al., 2014) that aim to increase local population productivity (Platt & Bever, 2009).

Dioecious plant species are characterised by sex dimorphism in their emission of herbivore‐induced plant volatiles, which is another factor that determines the outcome of VOC communication. In response to specialist aphids (*Uroleucon macolai*), male plants of the woody shrubs (*Baccharis salicifolia*) released volatile signals that induced resistance in both male and female receivers, whereas VOC signals from the female plants only induced resistance in female receiver plants (Moreira, Nell, Meza‐Lopez, Rasmann, & Mooney, 2018). Plant sex variation in the response to *U. macolai* feeding is reflected by the increase in pinocarvone emission that was five times higher in female plants than in male plants. Unlike male plants, females need to invest more resources in reproduction, and for this purpose, females have higher levels of induced defences (Nell et al., 2018) that are less costly to produce than constitutive defences (Agrawal, 2011). This can explain the dimorphism in sensitivity to herbivory‐induced volatile signals by male and female plants, where females need to respond faster and mount defences more strongly to ensure reproductive success.

The presence of certain heterospecific neighbours can modify the volatile emissions and induce defence in focal plants. For instance, red clover (*Trifolium pratense*) plants emitted significantly higher amounts of constitutive and herbivore‐induced volatiles when growing in communities with the orchard grass (*Dactylis glomerata*) than in plants growing together with forbs (*Geranium pratense*) (Kigathi, Weisser, Reichelt, Gershenzon, & Unsicker, 2019). Another study showed that invasive weed spotted knapweed (*Centaurea maculosa*) had the capacity to modulate growth and defence strategies in response to the presence of conspecific or heterospecific Idaho fescue (*Festuca idahoensis*) neighbours. When grown with heterospecific neighbours, *C. maculosa* allocated more resources towards growth, but when grown with conspecific neighbours, *C. maculosa* invested more resources to defence (Broz et al., 2010). It remains unknown whether VOCs or root exudates were responsible for such differences in the response of *C. maculosa* to conspecific or heterospecific neighbours.

Some herbivorous have insects developed the capacity to manipulate VOC emissions through the host plants and take advantage of the wrong receiver response. Thus, whiteflies (*Bemisia tabaci*) could deceive host plants to induce salicylic acid (SA)‐related defences while suppressing JA‐related defences and, through the release of volatile signals, could modify the quality of neighbouring plants for their offspring (Zhang et al., 2019). Instead of investing in defences against herbivorous insects, neighbouring plants invest in defence against pathogens, which is likely to make them defenceless when the next whitefly generation emerges.

### 2.6 Changes in volatile emission induced by abiotic stress interfere with plant VOC communication

Volatiles can play multifunctional roles as they have shown in their capacity to affect neighbouring plants and prime them against herbivore attack and abiotic stressors, to promote earlier flowering and to increase reproductive success under stressful situations (Cofer, Engelberth, & Engelberth, 2018; Landi et al., 2020). Frequent changes in abiotic conditions provide an important source of variability in plant communication at the community level, as they can trigger plant adaption responses and induce changes in the emission of volatiles (Tang, Valolahti, Kivimäenpää, Michelsen, & Rinnan, 2018) (Figure 2E–H). In response to cold stress, tea plants release nerolidol, which is absorbed and converted into glucoside and, consequently, improves cold stress tolerance (Zhao, Zhang, et al., 2020) (Figure 2E).

In addition to nerolidol, other volatile compounds such as geraniol, linalool and methyl salicylate, emitted from cold‐stressed plants, play a key role in priming cold tolerance (Zhao, Wang, et al., 2020). Global warming in the Arctic tundra affected dwarf birch (*Betula nana*) to significantly increase the amount of total terpenoids in response to herbivory, revealing a strong synergy between higher temperatures and herbivory (Li, Holst, Michelsen, & Rinnan, 2019). Plant adaption to fluctuations in temperature also occurred belowground, where the production of a root defence sesquiterpene lactone taraxinic acid β‐D glucopyranosyl ester of dandelion (*Taraxacum officinale*) covaried with the mean monthly temperature and the expected attack of a major root herbivore (*Melolontha melolontha*) (Huang, Bont, Hervé, Robert, & Erb, 2020). These unique plant responses to changes in temperature through the release of specific VOCs are particularly likely to be adaptive because plants adjust their traits in a timely manner to directly cope with stress and inform the neighbours about upcoming stresses.

Moreover, conditions of drought or high salinity cause to change their volatile emission (Caparrotta et al., 2018; Jardine et al., 2015; Vivaldo, Masi, Taiti, Caldarelli, & Mancuso, 2017) (Figure 2G,H). To test the effect of high salinity, a study identified 7,210 changes in gene expression under constant salinity stress and cross‐worked the volatile constituents. Changes in VOC composition, such as monoterpenes and isoprene release (Fernández‐Martínez et al., 2018), can protect the plant against abiotic stresses and help it cope with oxidative and thermal stress (Loreto, Pinelli, Manes, & Kollist, 2004; Penuelas, LlusiÀ, Asensio, & MunnÉ‐Bosch, 2005). In the presence of salt, tomatoes released more hydrophilic compounds and showed a decreased emission of 2‐decanone and alpha‐ionone, where most of the volatile emission‐related genes were repressed (Benjamin, Silcock, Leus, & Everett, 2012; Loreto & Schnitzler, 2010). These included β‐phellandrene synthase, allene oxide synthase, farnesyl pyrophosphate synthase, mevalonate kinase and geranyl pyrophosphate synthase. Several other pathways were induced by NaCl, including divinyl chlorophyllide and 8‐vinyl‐reductase (Zhang, Zeng, Chen, Sun, & Ma, 2018). Neighbouring plants can perceive salt‐stress‐induced volatile signals such as monoterpenes, isoprene and pentanal, and respond to upcoming stress by preparing in a timely manner (Caparrotta et al., 2018).

## 3 PLANT–PLANT COMMUNICATIONS IN THE COMPLEX VOLATILE ENVIRONMENT

Plant–plant communication is inherently complex as plants must distinguish between reliable cues or signals and determine plant fitness and performance in their environment, either by modulating defence response or by investing in reproduction and growth (Ninkovic et al., 2016; Ninkovic, Rensing, Dahlin, & Markovic, 2019; Pierik, Ballare, & Dicke, 2014) (Figures 1 and 2). Different groups of VOCs are involved in response to biotic stress such as herbivory (Ameye et al., 2018; Baldwin, 2010), mechanical damage, (Karban et al., 2014) pathogens (Hammerbacher et al., 2019) and touch (Markovic et al., 2019; Markovic, Glinwood, Olsson, & Ninkovic, 2014). Plants may also release a blend of specific VOCs when exposed to various abiotic stresses that include salinity (Caparrotta et al., 2018), ozone (Kanagendran, Pazouki, & Niinemets, 2018), temperature (Loreto, Pollastri, Fineschi, & Velikova, 2014) and light (Kegge et al., 2015). Higher concentrations of atmospheric pollutants such as ozone, nitrogen oxides and hydroxyl radicals can react with VOCs, degrading some of the active compounds and modifying the signal strength and fidelity (Blande, Holopainen, & Niinemets, 2014; Mofikoya, Kivimäenpää, et al., 2018). In this situation, the altered blend may be inactivated or it may provide different information to that of the volatile blend actually emitted by the plant (Figure 2F).

Degradation and formation of new products can alter the composition and ratio of key components in VOC profiles that reduce insect host location efficiency (Blande, Li, & Holopainen, 2011; Li, Blande, & Holopainen, 2016). All of these factors add a degree of complexity to the group of VOCs released as compared to the constitutive volatiles (cues), making the receiver response even more complicated and challenging. Complexity in the plant–plant interactions by VOCs is not only a matter of multiple chemical signals, but also a consequence of spatial and temporal variations in these signals (Giron‐Calva, Molina‐Torres, & Heil, 2012). For example, in dense stands, not all parts of a plant experience the exact same combination of VOC signals that can be dynamic over time (Douma, Ganzeveld, Unsicker, Boeckler, & Dicke, 2019). These variations in VOCs create a challenging task for plants, as they must respond only to important signals that point to a real threat. It remains unclear whether plants can accomplish this task without having negative fitness consequences.

## 4 PERCEPTION OF VOCS BY NEIGHBOURING PLANTS

**FIGURE 3:**
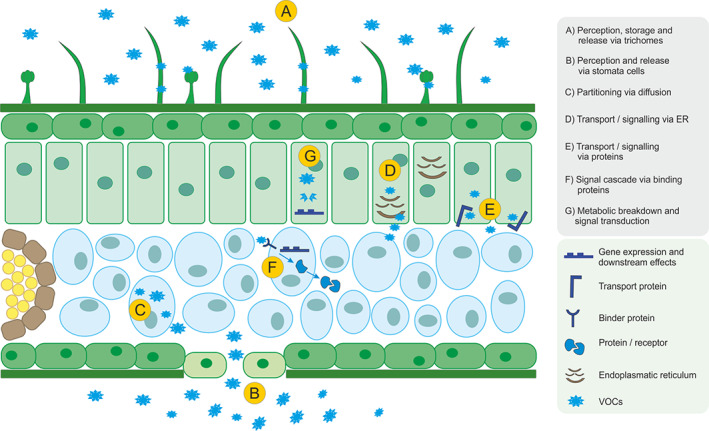
Plant perception mechanisms of VOCs: A, storage and release of VOCs via glandular trichomes; B, release and uptake of VOCs via stomata cells as part of perceptive and emission mechanisms; C, partitioning of VOCs via a bidirectional diffusion along a concentration gradient; D, nonvascular lipophilic transport via the endoplasmic reticulum (ER) during direct contact with the cell wall; E, VOCs or metabolite transport via specific transport proteins; F, perception of VOCs from specific receptors (extra or intracellular) with a resulting signalling cascade and gene expression; G, metabolic breakdown of VOCs resulting in fragments with signalling characteristics [Colour figure can be viewed at wileyonlinelibrary.com]

A rapid response to stress is a crucial factor in a plant's life. The perception of VOCs by plants is an essential process by which plants interact with one another. Although the exact mechanisms are largely unknown, recent research has shed light on some possible mechanisms, including hormonal pathways, specific structures and specialised proteins (Figure 3).

### 4.1 Specific plant structures of VOCs perception

The perception and uptake of VOCs from a metabolic pathway standpoint have attracted recent interest. It has been shown that VOCs can be taken up by the plant via the stomata and by adsorption through the leaf surface (Tani & Hewitt, 2009; Tani, Tobe, & Shimizu, 2010) (Figure 3B). These routes of entry have been measured by observing the uptake rates when the stomata were open or closed, showing that specific VOCs could be adsorbed through the leaf surface. Upon entry, VOCs underwent reduction and esterification, which increased their volatility and changed the profile upon rerelease. One example of this progress could be observed where VOCs underwent glycosylation and glutathionylation, which partially converted them into a non‐volatile compound. The newly produced (Z)‐3‐hexenyl vicianoside gave uninjured plants the ability to defend themselves against future herbivore attacks (Sugimoto et al., 2014).

One mechanism involved in this process has been shown to involve plant glandular trichomes (Figure 3A) that proved to be especially important for plant–plant interactions due to the rapid release of VOCs. Glandular trichomes are capable of storing high concentrations of chemicals, many of the VOCs, preventing them from re‐entering into the subcellular space where they could be toxic. These VOCs, released from the trichomes, can act as a signal for surrounding plants (Tissier, Morgan, & Dudareva, 2017), which result in a sudden spike in the concentration of VOCs that can be taken up and processed. Interestingly, this would provide a mechanism for the quick response of herbivore attacks without the timely upregulation of associated metabolic pathways. Glandular trichomes could also release stored VOCs after damage caused by strong winds or mechanical wounding. In this respect, we can reasonably assume that the neighbouring plants could benefit the most because they will get information about possible upcoming stress. This system would circumvent the need for a plant to first upregulate VOC‐dependent pathways to react to incoming signals. A study that examined trichome density and VOC emission rate in mountain birch (*Betula pubescens ssp*. *czerepanovii*) highlighted this possible mechanism during plant–plant interactions. Here, the emission rate of terpenoids was linked to trichome density in birch with an understorey of shrub (*Rhododendron tomentosum*) (Mofikoya, Miura, et al., 2018).

The transport of VOCs within the plant and between the cells has been shown to include a transport system via a nonvascular lipophilic pathway upon direct contact with the endoplasmic reticulum and the plasma membrane (Widhalm, Jaini, Morgan, & Dudareva, 2015) (Figure 3D). Another form of transportation can be the protein‐mediated movement (Samuels & McFarlane, 2012). These mechanisms of transport and upstream synthesis pose the problem of VOC hyperaccumulation within plant cells. To circumvent the toxic hyperaccumulation, a system of plasma membrane‐localised transporters has been observed, such as toxic compound extrusion pathways (Samuels & McFarlane, 2012). The ability of plants to have highly specific transport systems in place to shift VOCs and their metabolites through their cells and tissues shows a possibility for the perception of VOCs. A similar system could be used to gather information on the growth and stress status of surrounding plants.

VOCs can be partitioned between the intercellular space and the cell wall and, depending on their oil/water partition coefficients, diffuse into the cytosol (Matsui, 2016) (Figure 3C). In the cytosol, VOCs could then be metabolised, leading to downstream effects in the plant, depending on the nature and concentration of these metabolites. Through metabolic processes such as glycosylation and glutathionylation, the VOC concentration in the cytosol is lowered, which leads to an increase in the cytosolic uptake of further VOCs. This drastic increase in VOC metabolites, NADPH and glutathione, is reduced rapidly, strengthening the response of plants to oxidative stress. Furthermore, one metabolite (a VOC glycoside) has been shown to activate defence against herbivorous insects. A study by (Wenig et al., 2019) showed a feed‐forward loop involving systemic acquired resistance after monoterpene‐associated responses. This acquired resistance has been shown to be activated by SA‐mediated innate immune responses, which are essential in the plant perception of monoterpenes. Propagation of innate immune mechanisms provides a network that holds the potential for plant‐to‐plant induction of acquired resistance to herbivorous insects. Rapid response and downstream effects can jump to neighbouring plants and affect whole plant communities after an infection has occurred in a single individual. Esters of methyl salicylate (MeSA) and methyl jasmonate (MeJA) can diffuse into plant tissues and be converted by specialised esterases into SA and JA (Farmer & Ryan, 1990; Manosalva et al., 2010; Shulaev, Silverman, & Raskin, 1997; Wu, Wang, & Baldwin, 2008). Therefore, it is feasible that if a part of the metabolised VOCs is building blocks for downstream synthesis of other VOCs, these metabolites will then be part of the perceptive pathways in plant–plant communication (Figure 3G).

The mechanism involved in the perception of stress, such as bacterial or fungal pathogens or herbivorous insects attacks, can be found in the root system and is evident through membrane depolarisation. It has been shown that essential oils from peppermint (*Mentha piperita* L.) have the ability to increase membrane depolarisation (Maffei, Camusso, & Sacco, 2001). This change in the flux of ions across the plasma membrane can influence defence pathways within the plant, although not much is known about the precise mechanisms. Changing the rate of ion flux at certain points in time, that is, during an herbivore attack, might provide a quick response for plants to mount their defences.

### 4.2 Pathways related to VOC perception

Damage‐associated molecular patterns (DAMPs) provide a quick response to plant damage. When released from damaged plant cells, DAMPs activates the innate immune response of plants. In contrast to mammalian DAMPs, few binding receptors and pathways have been identified in plants. Herbivore‐induced plant volatiles can be classified as one type of DAMP that can be perceived by neighbouring plants (Duran‐Flores & Heil, 2016). The downstream effects and modes of action of these DAMPs include mitogen‐activated protein kinase activation, oxidative stress, membrane alteration and cell wall modifications (Figure 3E). After recognition of a threat, hormone signalling in chickpea was altered drastically with an 8.4% change in leaf transcriptome. The first to respond during DAMP recognition are JA, SA biosynthesis and ethylene‐related pathways (Pandey et al., 2017). These mechanisms provide a positive feedback loop in the receiving plant, as upregulation of JA bolsters the defence response as well as communication capabilities (Okada, Abe, & Arimura, 2014).

While DAMPs alter the VOC profile of receiving plants, they can also prime for future herbivore attacks. One of the better understood regulatory pathways includes systemin. It acts as a DAMP by binding to SR160 and then altering the expression of proteinase PI 1/2, which itself is relevant for a plant's defence during a herbivore attack (Duran‐Flores & Heil, 2016). Herbivore‐induced plant volatiles is established to act as a DAMP for intra‐ and interplant signalling (Heil, 2014) and to attract herbivore predators (Ninkovic & Pettersson, 2003). Not only DAMPs induce alterations in plant signalling pathways. A study by (Kutschera et al., 2019) showed that *Arabidopsis* can sense low‐complexity metabolites of Gram‐negative bacteria in the form of lipid A reduced elicitations. These metabolites have been shown to trigger an immune response in plants with further implications in interplant communication as described above. This underlines the precise regulatory pathways that plants use to alter their VOC emissions and, consequently, their communication.

This complexity of interplant communication is often observed at the plant level as well as at the molecular level. Recent research has shown genetic regulation during VOC perception. Using a heterologous pinII::GUS reporter system in *Arabidopsis thaliana*, it has been reported that certain terpenoid volatiles mediate a vast range of changes in the transcriptome (Godard, White, & Bohlmann, 2008). For this study, plants were exposed to either myrcene volatiles or to a blend of ocimene volatiles. Two major themes were identified: 1) the VOCs change several transcription factors, many of which are involved in the octadecanoid pathway, which are responsible for the biosynthesis of JA; 2) the VOCs induced an increased level of MeJA in tissues. Mutations in *aoc* and *coi1*, with both genes involved in the octadecanoid pathway, showed reduced sensitivity to VOCs (Godard et al., 2008). Interestingly, not only did these VOCs change the relative abundance of genes associated with transcription factors, but also in the membrane‐ and stress‐associated genes. This study showed that plant–plant communication and the perception of VOCs have an immense impact on plant's lifestyle. Specific alterations in the genome that affected defence strategies in the tobacco plants had wide‐reaching effects at the community level. Here, fields with few individuals genetically altered to produce an increased amount of defensive chemicals had a positive effect on disease emergence. These effects are probably beneficial due to a higher genetic diversity in the field (Schuman, Allmann, & Baldwin, 2015). This highlights the potential that can be found in the complex network of plant–plant communication (Subrahmaniam et al., 2018).

### 4.3 Specific binding proteins and regulation of VOC perception

Limited studies have explored the exact pathways and molecules by which VOCs are perceived by a plant. Understanding these would enable researchers to further investigate the regulatory mechanisms of growth and plant defences at the individual plant and community levels. Knowledge of the specific ways by which plants alter their emission of VOC before and after perception can provide deeper insights into the metabolic pathways that are essential for a plant community to thrive. An increased understanding of these mechanisms would enable discrimination between volatile cues and signals and their specified roles in plant–plant interactions. One protein involved in this VOC binding has recently been identified as a TOPLESS‐like protein (TPL) (Nagashima et al., 2019) (Figure 3F). The VOC involved in this study was β‐caryophyllene, which showed activity as a transcriptional co‐repressor. This shows that plant perception of VOCs is not only dependent on the structural mechanisms through membrane receptors, organs and metabolic pathways, but also on the underlying genetic regulation (Nagashima et al., 2019). The study system used tobacco plants and a BY‐2 cell culture. Cell culture experiments showed that overexpression of TPLs reduced the caryophyllene‐induced gene expression (Nagashima et al., 2019). The authors investigated three different groups of genes, *NtOsmotin* (a pathogenesis‐related gene), *NtODC* (a JA‐related gene) and *NtACIII* (an SA‐related gene) and tested 16 VOCs for their induction or inhibition properties. They found that each gene had a specific VOC with inducing capabilities_._ For a more specific understanding of induction, *NtOsmotin* was chosen for further analyses. β‐caryophyllene derivative‐linked beads were used to identify TPLs proteins, and two mechanisms of action have been proposed. The first is that β‐caryophyllene binds TPL, releasing an unknown co‐factor, which itself inhibits transcription factors when bound to TPL. The secondary mechanism by which β‐caryophyllene could induce expression of *NtOsmotin* is by binding TPL competitively, preventing it from binding to the unknown co‐factor (Nagashima et al., 2019).

### 4.4 Evolutionary aspects of plant–plant communication

From an evolutionary perspective, one of the oldest plant species is the bryophytes. The first evidence of plant–plant interactions via VOCs in non‐vascular plants was observed in a study showing that bryophytes can recognise their neighbours (Vicherová, Glinwood, Hájek, Šmilauer, & Ninkovic, 2020). Upon receiving VOCs from *Sphagnum flexuosum*, peatland moss (*Hamatocaulis vernicosus*) altered its growth pattern and emission of β‐cyclocitral to a degree six times higher than that of the negative control. This offered new insights into the evolution of plant–plant interactions via VOCs. Based on this study, it can be determined that VOCs are generally perceived as an essential tool in plant–plant communication and have been so for millions of years.

A long‐term strategy for the development of a plant's life is to provide the next generation with an advantage in terms of their specific surroundings. These exact evolutionary mechanisms are still under debate as this system is prone to false signalling, giving another surrounding plant a disadvantage. Attempts have been made to unravel VOC related signalling pathways in order to understand the complex networks of VOC emissions (Vivaldo et al., 2017). Investigating this complex network could allow us to understand relationships between plants related to evolutionary processes in a given environment (Vivaldo et al., 2017). Long‐term adaptation is not only observed in large plant communities, but also in localised communities. A genetic mechanism by which airborne plant–plant communication and ultraviolet light (UV‐C) irradiation activates DNA repetitive elements in *A. thaliana* has been shown (Xu et al., 2016). The expression levels of these elements are, under regular conditions, inhibited epigenetically by transcriptional gene silencing (TGS). This study provides an indication of how plant communication is regulated under artificial conditions, and this could be further investigated in a natural setting. These inhibition mechanisms were relieved by treatments with exogenous MeJA and MeSA. Furthermore, disrupting the biosynthesis of JA and SA showed evidence that these forms of airborne communication are involved in epigenetic responses, thus affecting the long‐term strategy of the plant (Xu et al., 2016). Methylation analysis found that the DNA sequences of TGS‐GUS experienced changes in DNA methylation at the CG, CHG, and CHH, DNA base sites in neighbouring plants, receiving signals via airborne plant–plant communication induced through UV‐C interplant communication (Xu et al., 2016). As previously discussed, plants have a rapid response when receiving VOCs when it comes to metabolic pathways and immediate defence strategies.

## 5 FUTURE PERSPECTIVES

In nature, plants are simultaneously or in sequence exposed to a broad array of individual VOC cues and signals or a specific combination of these. Owing to the complex ecological effects they induce, plants must respond only to crucial ones to avoid compromising their growth and defence. This makes regulatory mechanisms extremely challenging, taking into consideration the trade‐off between cost and benefits of investment into adaptation. In addition, one or more important signals can be cancelled by the other one, leading plants to make wrong and costly decisions. In this respect, long‐distance signals are much easier to hinder, cancel or mask, so the real messages they carry are hidden or diluted to the level of non‐important cues. It is unclear whether and how plants can cope with a diverse array of VOCs in their environment, and which specific cues/signals may have higher priority to respond to. Deciphering the language of within‐ and between‐species plant communication requires studies under realistic ecological settings in which plant adaption responses determine plant fitness and survival at the individual and community levels.

Recent studies on plant–plant communication by VOCs have revealed some metabolic pathways that plants activate to cope with biotic and abiotic stress. The molecular mechanisms of plant VOC perception remain largely unknown currently. Some topics have been addressed in recent years, showing that during plant–plant interactions, the regulatory mechanisms of perception are vastly complicated and highly specific, depending on the plant species, habitat and local plant community. It is clear that, from an evolutionary standpoint, VOC perception is a crucial tool for a plant to adjust its growth strategy and improve its fitness. Understanding the complex regulation of genetic and metabolic pathways could provide an important tool for commercial and sustainable agriculture. For this, further research must be conducted to understand the many factors that influence plant–plant interactions. Taking advantage of the natural mechanisms of how plants cope with biotic and abiotic stress would give us an edge in the rising demand for food production, especially since the genetic capacity for yield increase has been nearing its full potential. Studies so far have looked at the complex genetic regulation of VOC perception, mechanical structures and the area of proteomics to understand the metabolic pathways. Many open questions remain to be answered, and a general universal understanding of how different plants perceive and react to VOCs during plant–plant interactions remains to be found.

## CONFLICT OF INTEREST

The authors declare no conflict of interest.

## AUTHOR' CONTRIBUTIONS

VN conceived the idea and revised the manuscript. DM, MR and VN contributed equally to manuscript writing and drafting. DM and MR prepared the figures.
